# A Newly Developed Tolles–Lawson Compensation Model Accounting for Eddy-Current Dynamic Response

**DOI:** 10.3390/s26196338

**Published:** 2026-10-08

**Authors:** Peng Gu, Dong Gao, Chunjiang Bian, Minghui Zhu

**Affiliations:** 1 Key Laboratory of Electronics and Information Technology for Space Systems, National Space Science Center, Chinese Academy of Sciences, Beijing 100190, China; gupeng19@mails.ucas.ac.cn (P.G.); bianchunjiang@nssc.ac.cn (C.B.); zhuminghui20@mails.ucas.ac.cn (M.Z.); 2 University of the Chinese Academy of Sciences, Beijing 100190, China

**Keywords:** aeromagnetic compensation, cross-validation, eddy-current dynamic response, ridge regression, Tolles–Lawson model

## Abstract

Aeromagnetic compensation mitigates the aircraft-induced magnetic interference in airborne magnetometer measurements, thereby providing the measurement accuracy required by geomagnetic navigation and aeromagnetic surveying. The most widely used compensation framework, the conventional Tolles–Lawson (T-L) model, approximates the eddy-current magnetic field as an instantaneous linear response to the rate of change of the external magnetic field. Our analysis demonstrates that this assumption becomes invalid when the structural characteristic time scale is comparable to the aircraft maneuver time scale. We therefore generalize the eddy-current term into a first-order dynamic element, resulting in a dynamic eddy-current T-L model in which the eddy-current response is no longer instantaneous, while the 18-term design-matrix structure is preserved and no additional parameters are introduced. Utilizing a public flight dataset, we compare the two models on independent validation flight lines with a single calibration segment. Ridge regression on the Mag 4 channel yields a consistent and statistically significant improvement, where the median enhancement in the high-dynamic flight lines reaches +1.177 nT, approximately 70 times greater than that observed in regular survey lines. This benefit arises precisely under the high-dynamic flight conditions that are most critical for geomagnetic navigation, and its robustness is confirmed by dual-reference, vector-input, and regularization-parameter analyses.

## 1. Introduction

Geomagnetic navigation leverages the Earth’s magnetic field along with local magnetic anomalies to provide positional constraints [1], serving as a crucial alternative when GPS navigation is either degraded or unavailable. The navigation accuracy achievable with this technique depends directly on the accuracy of magnetometer measurements [2]. Airborne magnetometer data not only capture the geomagnetic signal but also incorporate magnetic interference originating from the platform structure, electromagnetic interference from onboard equipment, and other unmodeled disturbances. Given that the geomagnetic signal is relatively weak, magnetic interference from the platform structure commonly emerges as a significant factor that restricts navigation accuracy. Therefore, aeromagnetic compensation remains a fundamental challenge in the engineering applications of geomagnetic navigation.

The Tolles–Lawson (T-L) model is the most prevalent framework employed in aeromagnetic compensation [3]. This model decomposes aircraft-induced aeromagnetic interference into three components: permanent, induced, and eddy-current magnetic fields [4]. It then projects these interference components onto the sensitive direction of the magnetometer using 18 linear basis functions to establish a scalar compensation model [5]. Due to its clear structure, explicit physical interpretation, and straightforward implementation, the T-L model continues to serve as a foundational reference in aeromagnetic compensation research.

Research on the T-L model has predominantly pursued two avenues. The first avenue concentrates on the stability of coefficient estimation, primarily addressing issues of multicollinearity and ill-conditioning within the T-L design matrix. Zhao et al. demonstrated that the T-L model is frequently formulated as a linear regression problem [6]; however, the presence of multicollinearity among variables diminishes the accuracy of coefficient estimation. To mitigate the effects of multicollinearity, they constructed multiple models based on flight heading and utilized variable selection techniques. Building upon the aeromagnetic compensation challenge associated with multirotor unmanned aerial vehicles (UAVs), Qiao et al. identified that the T-L coefficient matrix is similarly impacted by multicollinearity and ill-conditioning [7]. They further highlighted that conventional principal component analysis (PCA) is particularly sensitive to noise, necessitating the implementation of robust principal component analysis to enhance compensation stability. The second avenue seeks to augment the capability of the T-L model in representing additional complex interference sources through the introduction of supplementary physical terms, nonlinear models, or data-driven structures. Du et al. proposed an extended aeromagnetic compensation model that integrates non-maneuver-related interference [8], including currents from onboard electrical systems, beacon lights, strobe lights, and control-surface motion, into the compensation framework. In furtherance of this, Zhang et al. addressed magnetic interference generated by currents in airborne electronic equipment [9]. They employed vector magnetometers to synthesize the total magnetic field and combined this data with singular spectrum analysis (SSA) to extract features related to current-induced magnetic interference. Subsequently, they developed a compensation model that incorporates conventional T-L terms, geomagnetic-gradient terms, and current-induced magnetic-interference terms. More recently, several studies have continued to advance both avenues. Yu et al. dynamically parameterized the compensation coefficients as first-order Fourier functions of the flight heading [10], Chen et al. introduced attitude-driven dynamic-enhancement terms into the eddy-current representation [11], and Li et al. modeled the geomagnetic field through Gaussian process regression to refine coefficient estimation [12], whereas Ye et al. combined Lasso regularization with Newton iteration to suppress multicollinearity in the design matrix [13].

Nevertheless, the physical modeling assumptions and applicability boundaries of the three T-L interference terms have been largely overlooked. This concern is particularly pertinent for the eddy-current term. Traditionally, the eddy-current magnetic field is mainly characterized as an instantaneous reaction to the rate of change of the external magnetic field. This inherently presumes that the response time of the conductive structure to fluctuations in the external field is negligible relative to the maneuver time scale. However, if the principal eddy-current pathways within an actual conductive structure exhibit significant build-up and decay processes, the eddy-current magnetic field will not only depend on the rate of change of the external field but may also demonstrate a distinct phase shift. In such scenarios, merely refining coefficient-estimation techniques or broadening the model cannot change the response form of the eddy-current term, leading to a mismatch between the T-L model structure and the actual response.

To tackle this issue, we propose a dynamic modification to the eddy-current term within the conventional T-L framework. The main contributions of this study are summarized as follows:

- Mechanism analysis: We methodically examine the formation mechanism of the eddy-current magnetic field through analysis of the magnetic diffusion equation and carefully identify the physical conditions under which the instantaneous rate response is inadequate.
- Dynamic model construction: We generalize the eddy-current term as a first-order dynamic element; utilizing zero-order-hold (ZOH) discretization, this element can be formulated as first-order recursive filtering applied to the eddy-current columns of the T-L design matrix, thus maintaining the 18-dimensional linear regression estimation structure.
- Simulation and flight validation: We conduct finite element method (FEM) simulations using a simplified three-compartment aircraft model to assess the influence of the structural characteristic time scale on the phase of the eddy-current magnetic field; employing a public flight dataset, we then carry out cross-flight validation with a single calibration segment and evaluate the effectiveness and robustness of the dynamic eddy-current T-L model under actual flight conditions.

In contrast to prior studies, which typically evaluate compensation performance on calibration flights collected from a single platform over a single survey area [10,11,12,13], the present validation employs a public flight dataset and generalizes across independent flight lines drawn from separate flights, thereby offering a more stringent and broadly representative assessment of the proposed model.

The remainder of this paper is organized as follows. Section 2 analyzes the formation mechanism of the eddy-current magnetic field and develops the dynamic eddy-current T-L model. Section 3 presents the FEM simulations of the eddy-current dynamic response. Section 4 reports the validation on the public flight dataset, including the comparison scheme, the results, and the robustness analyses. Section 5 concludes the paper.

## 2. Eddy-Current Mechanism Analysis and Modification of the T-L Model

### 2.1. Formation Mechanism of the Eddy-Current Magnetic Field

When a geomagnetic-navigation platform moves or when the external geomagnetic environment varies over time, the geomagnetic vector in the platform’s body frame also varies accordingly. For conductive structures, such as fuselage shells, frame constructs, and equipment housings, the magnetic flux through their localized cross-sections or closed loops undergoes corresponding changes. This variation induces currents, specifically eddy currents, within these structures or in proximity to their surfaces. These eddy currents generate an additional magnetic field, known as the eddy-current magnetic field. Among the three types of interference outlined by the conventional T-L model, the eddy-current magnetic field is distinguished by its direct correlation with the time-varying nature of the external field. Therefore, it demonstrates a pronounced temporal dependence and heightened sensitivity to external-field variation.

When employing the quasi-static approximation and neglecting the displacement current [14], the electromagnetic fields within a conductive structure satisfy the following equation:

(1) $$\left\{\begin{array}{cc} \nabla \times E & =-\frac{\partial B}{\partial t},\\ \nabla \times H & =J,\\ J & =\sigma E,\\ B & =\mu H, \end{array}\right.$$

where σ denotes the electrical conductivity, and μ is the magnetic permeability. Eliminating E and H in the above relations gives the magnetic diffusion equation [15]

(2) $$\frac{\partial B}{\partial t}=\frac{1}{\mu \sigma }\nabla^{2}B.$$

This equation illustrates that the evolution of the magnetic field within a conductive structure behaves fundamentally as a diffusion process, which is regulated by the diffusion coefficient. When focusing solely on the time required for the magnetic field to penetrate the structural thickness, the associated characteristic time scale can be approximated by

(3) $$\tau_{⊥}\approx \mu \sigma h^{2},$$

where *h* denotes the effective structural thickness. For common aluminum-alloy structures with centimeter-scale thickness, $\tau_{⊥}$ is on the order of milliseconds. In the low-frequency range corresponding to platform maneuvers, this time scale is significantly shorter than both the maneuver time scale and the magnetometer sampling period. Therefore, through-thickness magnetic diffusion can be regarded as a fast response to variations in the external field. Under this approximation, the magnetic flux through an equivalent structural loop, the induced electromotive force, the equivalent loop voltage, and the eddy current can be expressed by

(4) $$\Phi \left(t\right)=\int_{S}B\left(t\right)\cdot dS\approx SB_{n}\left(t\right),\;B_{n}\left(t\right)=B\left(t\right)\cdot n,$$

(5) $$\oint_{\partial S}E\cdot dl=-\frac{d\Phi }{dt}\approx -S\frac{dB_{n}\left(t\right)}{dt},$$

(6) $$U_{eq}=El,$$

(7) $$I_{eddy}=\frac{U_{eq}}{R}\propto \frac{S}{R}\frac{dB_{n}}{dt},$$

where *S* represents the loop area, n denotes the unit vector along the loop normal, B is the magnetic field passing through the loop, ∂S denotes the curve boundary, *l* is the loop perimeter, and *R* is the loop resistance.

According to the Biot–Savart law [16], the magnetic field generated by eddy currents at a given observation point is directly proportional to the current flowing through the loop. Therefore, the expression for the eddy-current magnetic field can be formulated as

(8) $$B_{e}=K_{e}{\dot{B}}_{o},$$

where $B_{o}$ denotes the external magnetic field, and $K_{e}$ represents a coefficient matrix that accounts for the shape, size, electrical conductivity, and observation-point location of the conductive structure. Because the local principal-axis coordinate system of the structure is generally not aligned with the platform body frame, $K_{e}$ is not a diagonal matrix, representing a 3×3 linear coupling matrix. Equation (8) represents the instantaneous rate-response form used for the eddy-current term in the conventional T-L model.

However, in large-scale structures such as the fuselage shell, internal eddy currents do not exclusively manifest as through-thickness diffusion. A more prevalent case involves the formation of large-scale circulating currents near the surface of the fuselage shell. Under these circumstances, the characteristic time scale should be estimated in terms of order of magnitude as

(9) $$\tau_{\|}\approx \mu \sigma rh,$$

where *r* denotes the fuselage-shell radius. For instance, for an aluminum-alloy fuselage shell of radius 0.3 m and wall thickness 0.02 m, $\tau_{\|}$ is approximately predicted as 0.26 s. This value is already comparable to the platform’s maneuver time scale. In this situation, the response of the eddy-current magnetic field to external-field variation exhibits a non-negligible phase lag, and the instantaneous rate-response form in Equation (8) would no longer be applicable.

### 2.2. First-Order Dynamic Modification of the Eddy-Current Term

Equation (8) can be generalized by introducing a first-order dynamic element as follows:

(10) $$\tau {\dot{B}}_{e}+B_{e}=K_{e}{\dot{B}}_{o},$$

where τ denotes the time constant. When τ is much smaller than the platform’s maneuver time scale, the first term on the left-hand side of Equation (10) becomes negligible. The equation then reduces to the instantaneous rate-response form given by Equation (8). This indicates that the eddy-current term in the conventional T-L model can be regarded as the limiting case of Equation (10) as τ→0.

Compared with Equation (8), Equation (10) only adds a low-pass dynamical element in the time domain to the original instantaneous mapping, whereas the linear relationship between $B_{e}$ and $B_{o}$, as well as the 3×3 matrix structure of $K_{e}$, is preserved. As a result, the modified eddy-current term can be directly incorporated into the conventional 18-dimensional T-L basis-function framework.

### 2.3. Dynamic Eddy-Current T-L Model

The conventional T-L model represents the scalar projection of platform-induced magnetic interference along the magnetometer sensitive direction, $b_{int}$, as a linear combination of 18 basis functions in the following form:

(11) $$b_{int}=\sum_{i=1}^{18}\beta_{i}a_{i}=a^{T}\beta ,$$

in which $a_{1}$–$a_{3}$, $a_{4}$–$a_{9}$, and $a_{10}$–$a_{18}$ in order are associated with the permanent-field, induced-field, and eddy-current terms. Further, the terms $a_{10}$–$a_{18}$ can be derived from bilinear combinations of the direction cosines and the time derivatives of the external-field components. These correspond to the scalar expansion of the vector rate response $K_{e}{\dot{B}}_{o}$ in nine coupling channels.

Applying Equation (10) to the eddy-current term yields the dynamic eddy-current T-L model as follows:

(12) $$b_{int}\left(t\right)=\sum_{i=1}^{9}\beta_{i}a_{i}\left(t\right)+b_{e}\left(t\right),\;\tau {\dot{b}}_{e}\left(t\right)+b_{e}\left(t\right)=\sum_{j=10}^{18}\beta_{j}a_{j}\left(t\right).$$

The eddy-current channel components in Equation (11) are initially processed through an identical first-order filter before being linearly combined to derive $b_{e}$. The other two interference terms maintain their form as described in the conventional T-L model.

In addition, application of zero-order-hold (ZOH) discretization to Equation (12) with the sampling period $\Delta t=1/f_{s}$ leads to the following recursive relation:

(13) $$b_{e}\left[k\right]=\alpha b_{e}\left[k-1\right]+\left(1-\alpha \right)\sum_{j=10}^{18}\beta_{j}a_{j}\left[k\right];\;\alpha =\exp\left(-\Delta t/\tau \right).$$

For τ>0, α always lies within the interval (0,1). This indicates that the discrete system remains stable for any value of the ratio Δt/τ.

Owing to the linearity of first-order infinite impulse response (IIR) filtering, Equation (13) can be equivalently rewritten as

(14) $$b_{e}\left[k\right]=\sum_{j=10}^{18}\beta_{j}a_{j}^{\prime }\left[k\right];\;a_{j}^{\prime }\left[k\right]=\alpha a_{j}^{\prime }\left[k-1\right]+\left(1-\alpha \right)a_{j}\left[k\right].$$

Thus, the design matrix of the dynamic eddy-current T-L model can be derived by first applying column-wise first-order recursive filtering to columns 10–18 of the conventional T-L design matrix, followed by the same linear combination as in Equation (11). Let the filtered design matrix be denoted as $A^{\prime }=\left[A_{1:9},A_{10:18}^{\prime }\right]$. Its dimensions are consistent with those of the conventional T-L design matrix, requiring no additional parameters for estimation; hence, coefficient estimation in the dynamic eddy-current T-L model remains an 18-dimensional linear regression problem. This approach retains the same estimation structure as the conventional T-L model. The primary distinction lies in whether the eddy-current columns of the design matrix undergo first-order filtering before coefficient estimation.

### 2.4. Coefficient Estimation and Effective Time Constant

Because the dynamic eddy-current T-L model retains the same estimation structure as the conventional T-L model, any magnetic compensation algorithm developed for 18-dimensional linear regression can be applied directly. The only additional tunable parameter is the time constant τ of the first-order recursive filter. Given that conductive structures of varying scales, such as thin-walled fuselage shells, equipment housings, and internal frames, may correspond to distinct values of τ, the actual eddy-current dynamic response is a superposition of multiple first-order elements. Since the model introduces only one first-order element, the estimated parameter reflects an optimal effective value that encapsulates multiple time constants τ into a single first-order element. This value is represented by $\tau_{e}$, which signifies the dominant time scale of the eddy-current dynamic response within the present model, rather than the true time constant of any individual loop.

The value of $\tau_{e}$ can be rationally chosen from a preset search grid T using blocked *K*-fold cross-validation, where T is approximately constrained by the physical definition of $\tau_{\|}$,

(15) $$\tau_{e}=\underset{\tau_{e}\in T}{arg min}\frac{1}{K}\sum_{k=1}^{K}{∥y_{f}^{\left(k\right)}-X_{f}^{\left(k\right)}\beta^{\left(-k\right)}∥}^{2},$$

where (k) is the *k*-th validation subsegment, $y_{f}$ denotes the band-pass-filtered target signal, $X_{f}$ represents the band-pass-filtered design matrix corresponding to a candidate $\tau_{e}$, and $\beta^{\left(-k\right)}$ denotes the coefficient estimate fitted from the remaining subsegments after excluding the *k*-th subsegment. Blocked partitioning is preferred over random partitioning to maintain the local correlation structure inherent in time series data [17]. This approach helps to prevent the underestimation of cross-validation error that can arise when random sampling is applied to autocorrelated samples.

Since *K* is a single-digit number, and each fold involves only an 18-dimensional linear regression problem, the computational cost of the dynamic eddy-current T-L model remains comparable to that of the conventional T-L model.

## 3. Simulation of the Eddy-Current Dynamic Response

This section employs a simplified three-compartment aircraft model to validate the eddy-current dynamic effect previously discussed, utilizing simulation techniques. The finite element simulations reported in this section are performed using COMSOL Multiphysics v6.1 (COMSOL AB, Stockholm, Sweden). To streamline the analysis, the simulation is performed under a unidirectional external magnetic field excitation at a constant frequency and amplitude. This analysis investigates how the characteristic time scale of the conductive structure influences the phase of the eddy-current magnetic field.

### 3.1. Simplified Model and Simulation Setup

The simplified model has been illustrated in Figure 1. It comprises a hollow cylinder topped with a hemispherical nose, featuring a total length of 2 m, an outer diameter of 0.5 m, and a wall thickness of 0.01 m. Two connectors are positioned along the axial direction of the fuselage shell, creating distinct internal compartments: front, middle, and rear. To effectively localize the source of the eddy-current magnetic field to internal structural components that are amenable to analysis, the fuselage shell is represented as fiberglass-reinforced plastic (FRP), characterized by its negligible electrical conductivity.

Three ring-shaped structural components, designated as A, B, and C, are arranged within the compartments to represent common large metallic elements found in the platform. Each component possesses identical geometric parameters, featuring a major radius of 0.065 m and a minor radius of 0.015 m. The normal directions of these components are aligned with the *x*-, *y*-, and *z*-axes of the platform, respectively. An external excitation in the form of a sinusoidal time-varying uniform magnetic field is applied along the *x*-direction, mainly characterized by a fixed frequency and amplitude. The observation point is established at (0.9,0,0), allowing for the output measurement of the three-component time-varying eddy-current magnetic field at this specified location.

The geometric parameters are chosen to be representative of a small fixed-wing survey platform rather than to replicate a specific aircraft: the 2 m total length and the 0.5 m outer diameter correspond to the overall scale of such a fuselage, and the ring dimensions are comparable to those of common large metallic components inside the cabins. The frequency of the external excitation is set to 1 Hz so that the excitation period is comparable to the time scale of typical platform maneuvers, and the characteristic time scale is swept by varying the electrical conductivity of the rings, as described in Section 3.2. Accordingly, the simulation is intended to illustrate the mechanism of the eddy-current dynamic response rather than to reproduce any particular aircraft structure.

### 3.2. Parametric Sweep of the Characteristic Time Scale

According to Equation (9), when the geometric parameters and the magnetic permeability are held constant, $\tau_{\|}$ can be manipulated by varying the electrical conductivity σ. In this section, $\tau_{\|}$ is varied from 0.008 s to 1.6 s, which spans approximately two orders of magnitude around the estimated value of 0.26 s for a typical aluminum-alloy fuselage shell. The direction, frequency, and amplitude of the external excitation remain unchanged throughout the parametric sweep. The response curves for the three components of the eddy-current magnetic field at (0.9,0,0) are illustrated in Figure 2.

The results indicate that when $\tau_{\|}$ is on the millisecond scale or below, the three-component responses closely align in phase with the rate of change of the external field. This observation is consistent with the instantaneous rate-response form described by Equation (8). As $\tau_{\|}$ increases to a scale comparable to the excitation period, the three-component responses demonstrate a distinct phase lag with respect to the rate of change of the external field. With further increases in $\tau_{\|}$, this phase lag becomes more accentuated. These findings suggest that variations in $\tau_{\|}$ significantly influence the phase of the eddy-current magnetic field. This behavior aligns with the $\tau_{\|}$-dependent characteristics anticipated from the first-order dynamic element outlined in Equation (10). Thus, it is evident that when the characteristic time scale of a conductive structure approaches the maneuver time scale, the instantaneous rate-response formulation of the eddy-current term in the conventional T-L model is insufficient to accurately represent the actual phase relationship. As a result, the first-order dynamic representation provided by Equation (10) is essential as a corrective measure.

## 4. Validation of the Dynamic Eddy-Current T-L Model

Mechanism analysis and FEM simulations have demonstrated that when the characteristic time scale of a conductor aligns with the maneuver time scale, the instantaneous rate-response representation of the eddy-current term in the conventional T-L model becomes inadequate for accurately capturing the actual behavior of the eddy-current magnetic field. Therefore, the incorporation of the first-order dynamic element, as indicated in Equation (10), becomes essential. However, the FEM simulations are limited to a single case and do not explore the transferability across varying operational conditions. This section leverages publicly available flight data to estimate the coefficients of the dynamic eddy-current T-L model from an independent calibration segment. The prediction model is subsequently applied to multiple independent validation flight paths to assess its compensation performance. The primary objective is to examine whether first-order recursive filtering can maintain a stable compensation gain under cross-flight conditions.

### 4.1. Dataset and Experimental Setup

The experimental data utilized in this study are sourced from the publicly available flight dataset collaboratively released by the DAF-MIT AI Accelerator and Sander Geophysics Ltd. (SGL, Ottawa, ON, Canada) [18]. This dataset comprises six flight files, identified as Flt1002–Flt1007, sampled at a frequency of 10 Hz, and encompasses three distinct regions: Eastern Ontario, Renfrew, and the Figure of Merit (FOM). Notably, Flux A is completely absent in Flt1002, and the Mag 2 channel experiences signal dropouts at headings near 180°; therefore, these elements are excluded from the evaluation. The compensation targets are confined to Mag 3, Mag 4, and Mag 5. In the primary experiment, Flux D is employed as the default vector input, whereas Flux B and Flux C are designated for robustness testing. The layout of the airborne magnetometer is illustrated in Figure 3, and the experimental configuration is summarized in Table 1.

The flight data were collected in the summer of 2020 near Ottawa, Canada, using a Cessna Grand Caravan (Textron Aviation Inc., Wichita, KS, USA) operated by SGL [19]. All five scalar channels (Mag 1–Mag 5) are measured by optically pumped, split-beam cesium-vapor magnetometers, with a sensitivity of 0.005 nT and a sensor noise level below 0.02 nT as reported by the operator [20], although their manufacturer and model are not disclosed in the dataset documentation; Mag 1 is mounted at the end of a tail stinger, and Mag 2–Mag 5 are mounted in the front cabin, in the middle cabin, on the rear-cabin floor, and on the rear-cabin ceiling, respectively [19]. Flux A–Flux D are three-axis fluxgate vector magnetometers located in the middle cabin, at the base of the tail stinger, and on the port and starboard sides of the rear cabin, respectively; their exact model is not disclosed in the dataset documentation. In the paired comparisons of this study, however, the conventional and the dynamic models are constructed from the same fluxgate measurements, so the comparison results are not affected by the absolute calibration of the fluxgate sensors. Attitude information is provided by the three-axis gimballed inertial navigation system of the operator’s AIRGrav airborne gravimeter (Sander Geophysics Ltd., Ottawa, ON, Canada) [18]. The reference channel mag_1_c was produced from the Mag 1 measurement by SGL using its professional compensation procedure during the 2020 survey campaign, and the dataset also provides the diurnal variation measured by a ground-based reference magnetometer together with the International Geomagnetic Reference Field (IGRF) correction, both of which enter the map-based reference baseline.

Free-fly and transit operating conditions typically involve more pronounced attitude variations and increased rates of geomagnetic-field variation; consequently, these conditions are more likely to yield identifiable dynamic effects. Based on the flight types outlined in the official dataset description, all validation flight lines are categorized into two subsets as follows: (i) five free-fly and transit flight lines, including Flt1003/1003.02, Flt1003/1003.07, Flt1003/1003.08, Flt1007/1007.02, and Flt1007/1007.06, are designated as the high-dynamic subset; (ii) the remaining 27 Perth survey lines are classified within the regular-survey-line subset.

### 4.2. Comparison Scheme and Evaluation Metrics

We employ ordinary least squares (OLS), ridge regression (Ridge), and truncated singular value decomposition (TSVD) for coefficient estimation [21,22]. For each algorithm, a paired comparison is conducted between the conventional T-L model and the dynamic eddy-current T-L model. This design assesses not only the effectiveness of first-order recursive filtering but also its dependence on the chosen estimation algorithm.

All three algorithms utilize identical preprocessing parameters, which include a sampling rate of 10 Hz, a 0.1–0.9 Hz band-pass filter, a fourth-order Butterworth filter, and the removal of 20 samples from each end of the dataset. The Ridge regularization parameter is set to λ=0.025, consistent with the official example of MagNav.jl (https://github.com/MIT-AI-Accelerator/MagNav.jl, accessed on 14 August 2026). The TSVD truncation rank is selected from one to 18 utilizing five-fold blocked cross-validation. The effective time constant $\tau_{e}$ for first-order recursive filtering is appropriately selected, independently for each channel and algorithm, from a 17-point discrete grid spanning 0.01–2.0 s (denser near 1 s), using five-fold blocked cross-validation according to Equation (15). The sensitivity of this selection is examined in Section 4.3.1 and Section 4.4.3.

The evaluation metric is mainly constructed based on per-line paired comparisons of residual standard deviations (STDs). Let us take $\sigma_{s}$ and $\sigma_{d}$ as the residual standard deviations of a given flight line under the conventional T-L model and the dynamic eddy-current T-L model, respectively. In addition, the paired improvement is defined as $\Delta =\sigma_{s}-\sigma_{d}$, where a positive value indicates that the dynamic eddy-current T-L model outperforms the conventional T-L model on that flight line. Subsequently, five key statistics are reported for the overall evaluation: the median, the mean, the 95% bootstrap confidence interval of the median, the positive rate, and the one-sided Wilcoxon signed-rank test *p*-value [23,24]. The median reflects the improvement of a representative flight line, while the mean indicates the average level of improvement. The positive rate quantifies the proportion of flight lines that demonstrate improvement, and the *p*-value assesses whether the paired improvements exhibit a systematic bias towards the positive direction.

### 4.3. Results and Discussion

#### 4.3.1. Effective Time Constant Selection

Figure 4 illustrates the cross-validation error curves for the three compensation channels under Ridge, whose $\tau_{e}$ values are set as 0.01 s, 1.10 s, and 0.02 s. Among them, only the Mag 4 result clearly deviates from the lower bound near zero.

The $\tau_{e}$ values selected for each channel under OLS and TSVD are listed in Table 2. As is seen, most of these values remain at the lower grid bound of 0.01 s.

#### 4.3.2. Coupling Between the Dynamic Element and the Estimation Algorithm

The paired comparison results for the three algorithms across three channels are presented in Table 3. Notably, except for Ridge on Mag 4, most groups exhibit median and mean improvements that are nearly zero. In these near-zero instances, the signs primarily reflect rounding-level perturbations and lack meaningful physical interpretation. The corresponding extreme positive rates, such as 97% or 6%, are merely statistical artifacts arising from small perturbations around zero and thus warrant no further interpretation.

The only statistically significant improvement is observed for Ridge on the Mag 4 channel, yielding a median enhancement of +0.020 nT, accompanied by a one-sided Wilcoxon signed-rank test *p*-value of 0.002, and a positive rate of 68.8%. The underperformance of OLS and TSVD across the channels can primarily be attributed to the magnitude of the coefficients. In the same Mag 4 calibration segment, the norm of the 18-dimensional coefficient vector estimated by OLS is approximately $3.67\times 10^{4}$. TSVD selects a rank of 17, resulting in a coefficient norm that is also approximately equal to $3.66\times 10^{4}$, which is comparable to that of OLS. Under λ=0.025, the coefficient norm of Ridge is approximately equal to $1.27\times 10^{3}$, which is over an order of magnitude smaller than those obtained from OLS and TSVD. When the estimation algorithm permits large coefficients, these coefficients can capture a portion of the residual associated with eddy-current dynamics in the least-squares sense. As a result, the remaining residual lacks sufficient signal for cross-validation to identify a $\tau_{e}$ that deviates from the lower bound. Conversely, by constraining the coefficients to a more reasonable magnitude, Ridge allows the lagged component of eddy-current dynamics to manifest as an independent signal in the residuals. This enables cross-validation to reliably identify a $\tau_{e}$ that deviates from the lower bound.

The primary reason Ridge does not enhance Mag 3 and Mag 5 is mainly attributed to the weak eddy-current response at their respective cabin locations. Mag 3 is situated near the inertial navigation system (INS) in the middle cabin at (−1.28,−0.36,0), far removed from the large-scale conductive structures present in the rear cabin. In contrast, Mag 5 is located on the rear-cabin ceiling at (−3.79,0,1.20), whereas Mag 4 benefits from being surrounded by the fuselage skin, internal conductive structures, and equipment cabinets. Although both Mag 5 and Mag 4 are located within the rear cabin, the ceiling location of Mag 5 results in a different local conductive structure geometry compared to Mag 4. These factors contribute to various observable strengths of large-scale circulating-current responses across the three channels.

For first-order recursive filtering to function stably under cross-flight conditions, two essential criteria must be met: coefficient-magnitude constraints imposed by the estimation algorithm and a conductive environment that can facilitate a substantial eddy-current response at the location of the compensation channel. Ridge provides the necessary constraints, whereas OLS and TSVD do not provide such a constraint in this dataset. Mag 4 meets the latter requirement, whereas Mag 3 and Mag 5 do not under the magnetometer layout of this dataset. Improvement in the dynamic eddy-current T-L model is only statistically detectable when both conditions are satisfied. Consequently, the subsequent analysis focuses on Ridge applied to the Mag 4 channel, incorporating the dynamic eddy-current modification, which is referred to as D-Ridge.

#### 4.3.3. Stratification by Operating Condition

The paired improvements for the 32 validation flight lines are ranked in Figure 5. The positive improvements are predominantly concentrated in five flight lines: Flt1003/1003.02, Flt1007/1007.02, Flt1007/1007.06, Flt1003/1003.07, and Flt1003/1003.08. Each of these improvements exceeds +0.3 nT, and all are classified within the high-dynamic subset. In contrast, the majority of the remaining flight lines’ improvements are distributed around ±0.05 nT, closely approaching the limits of numerical precision.

Table 4 summarizes the paired improvements by subset. The high-dynamic subset demonstrates a median improvement of +1.177 nT, which is nearly 70 times greater than the improvement observed in the regular-survey-line subset (+0.017 nT). Due to its larger sample size, the regular-survey-line subset yields a one-sided Wilcoxon signed-rank test *p*-value of 0.024, indicating statistical significance. Moreover, the high-dynamic subset produces a *p*-value of 0.031, which likewise achieves the 0.05 significance level, and its improvement magnitude is clearly greater than that of the regular-survey-line subset. When both subsets are combined, the analysis of all 32 flight lines results in a median improvement of +0.020 nT and p=0.002. The directional difference between the two subsets aligns with the mechanism analysis. Free-fly and transit flight lines exhibit stronger attitude variations and increased rates of external-field variation, making them more likely to induce the dynamic response of eddy currents in conductive structures. In contrast, regular survey lines approach a quasi-steady flight condition, for which the instantaneous rate approximation is generally adequate.

To quantify this interpretation, the eddy-current excitation strength of each validation flight line is computed as the root-mean-square (RMS) value of the band-pass-filtered rate of change of the vector magnetic field measured by Flux D; this rate of change is exactly the quantity that drives the eddy-current columns of the design matrix. Table 5 summarizes the results. The median excitation of the high-dynamic subset is approximately 1.7 times that of the regular-survey-line subset. Moreover, across all 32 lines, the per-line excitation is positively correlated with the per-line improvement, with a Spearman rank-correlation coefficient of ρ=0.42 (p=0.018); the positive ρ indicates that flight lines with stronger excitation tend to obtain larger improvements, and the *p*-value indicates that this association is statistically significant. The concentration of the improvement in the five high-dynamic flight lines is therefore an expected consequence of the excitation-dependent nature of the eddy-current dynamic response rather than an indication of limited applicability: on regular survey lines the eddy-current dynamics are only weakly excited, the instantaneous rate approximation is already adequate, and the attainable improvement is intrinsically small.

Figure 6 illustrates the paired comparison results for the three flight lines with the most significant attitude variations in the high-dynamic subset: Flt1003/1003.02, Flt1007/1007.02, and Flt1007/1007.06, which exhibit improvements of +1.390 nT, +1.260 nT, and +1.177 nT, respectively.

Figure 7 illustrates a comparison of the residual time series derived from a representative survey line and a representative high-dynamic line exhibiting significant attitude variation. For the survey line, the residual time series of D-Ridge and Ridge nearly coincide; nevertheless, on Flt1003/1003.07, D-Ridge demonstrates marked improvement over Ridge across several time intervals.

### 4.4. Robustness Analysis

#### 4.4.1. Dual-Baseline Consistency

The official dataset example utilizes the compensated Mag 1 measurement as the primary reference baseline. However, an independent map-based reference can also be derived from a magnetic anomaly map. Consequently, it is essential to evaluate whether the conclusion drawn above is influenced by the selection of the reference baseline. Table 6 presents a comparison of the median improvements across the three channels using the two different reference baselines.

For Mag 4, the median improvements observed under the two reference baselines are +0.020 nT and +0.024 nT, respectively, demonstrating a close alignment between the two values. The MeanAbsDiff is predicted to be only equal to 0.0071 nT, while the MaxAbsDiff is estimated to be only equal to 0.0625 nT. These results suggest that the conclusions pertaining to the Mag 4 improvement are robust, independent of the chosen reference baseline. Whether utilizing the map-based reference or the Mag 1 reference, the final conclusions remain unchanged.

#### 4.4.2. Vector-Input Sensitivity

The primary experiment employs Flux D as the default vector input. Should the conclusion drawn above apply solely to a single vector input, its physical validity would be deemed insufficient. Therefore, the comprehensive solution process of the dynamic eddy-current T-L model is executed again for Mag 4, utilizing Flux B, Flux C, and Flux D in succession, and their corresponding results are given in Table 7.

The $\tau_{e}$ values corresponding to the three vector inputs fall within the range of 0.8–1.3 s. This indicates that the clear deviation of $\tau_{e}$ from the lower bound does not rely on the choice of vector input. However, the paired test utilizing Flux D achieves the strongest statistical significance. While the median improvements for Flux B and Flux C are also positive, the statistical evidence for these two is comparatively weaker. This discrepancy can be attributed to the distinct installation locations of the three vector magnetometers. Flux B is positioned at the base of the tail stinger (−8.92,0,0.96), which is the farthest from the dominant conductive structures in the rear cabin. Conversely, Flux C (−4.06,0.42,0) and Flux D (−4.06,−0.42,0) are both situated within the rear cabin and have the same lateral distance of 0.42 m from Mag 4 (−3.53,0,0). However, they are located on opposite sides of Mag 4, leading to differing coupling with the asymmetric conductive structures present inside the rear cabin. This disparity elucidates the difference in statistical significance among the three vector inputs. Nonetheless, the order of magnitude of $\tau_{e}$ and the sign of the median improvement remains consistent across all three inputs.

#### 4.4.3. Regularization-Parameter Sensitivity

The main experiment utilized a specific value λ=0.025, consistent with the official example provided. This value is not treated as a freely tunable parameter for the present dataset. To assess how sensitive our conclusions are to this choice of λ, we select 10 logarithmically spaced λ values in the range of 0.0025 to 0.158. For each of these values, we repeat the complete solution procedure of the dynamic eddy-current T-L model. The obtained results are now demonstrated in Figure 8.

Figure 8a–d display the effects of λ on the overall median improvement, the *p*-value, the subset-level median improvement, and the cross-validation-selected $\tau_{e}$. The median (Δ) monotonically lessens with λ, from +0.045 nT at λ=0.0025 to +0.003 nT at λ=0.158. In addition, the one-sided Wilcoxon *p*-values are less than 0.05 for λ-values from 0.006282 to 0.158 within the tested grid, forming a U-shaped curve with the minimum at λ=0.040(p=0.001). Further examination of the subset-based results reveals that the median (Δ) of the high-dynamic subset is substantially larger than that of the regular-survey-line subset for all λ values, by approximately a factor of 70. This is clearly indicative of the fact that the phenomenon that the dynamic gain is concentrated in high-dynamic operating conditions remains stable in the presence of λ variation. The cross-validation-selected $\tau_{e}$ exhibits an inverse dependence on λ. In general, a smaller λ leads to a larger $\tau_{e}$. In the case of λ=0.0025, $\tau_{e}$ reaches the upper grid bound of 2.0 s, while for λ=0.158, it reduces to 0.3 s.

First, in the case of λ=0.0025, the median (Δ) reaches +0.045 nT, which is nearly twice that obtained at λ=0.025; however, the *p*-value rises to 0.074 and therefore the statistical significance is lost. This means that a smaller λ improves the median value but does so at the cost of statistical robustness. Second, the significance interval λ∈[0.006,0.16] covers more than one order of magnitude around 0.025. This is clearly indicative of the fact that λ=0.025 is not a finely tuned special solution. Considering the median (Δ), the *p*-value, and the Flux-input consistency, λ=0.025 provides an acceptable balance between physical consistency and statistical robustness.

## 5. Concluding Remarks

Starting from the magnetic diffusion mechanism of conductive structures, this study methodically explored the instantaneous rate-response assumption associated with the eddy-current term in the conventional T-L model. Our findings indicate that when the structural characteristic time scale is considerably smaller than the time scale of external-field variation, the eddy-current magnetic field can be approximated as an instantaneous linear response to the rate of change of the external magnetic field. However, in the context of large-scale conductive circulating currents in fuselage-shell structures, the characteristic time scale may be comparable to the aircraft maneuvering process. In this case, the eddy-current response exhibits a notable phase lag, rendering the conventional T-L eddy-current term potentially inadequate. To address this limitation, we introduce a first-order dynamic element into the eddy-current term.

The validation on public flight data reveals that the dynamic eddy-current modification is not universally effective across all channels, algorithms, and flight lines. A statistically detectable improvement is predominantly observed in the combination of the Mag 4 channel and Ridge. Over 32 independent validation flight lines, the overall median improvement is reported to be +0.020 nT, and the one-sided Wilcoxon signed-rank test gives p=0.002. After stratification by operating condition, the high-dynamic subset demonstrates a median improvement of +1.177 nT, approximately 70 times greater than that observed in the regular-survey-line subset.

The magnitudes of the coefficients and the placements of the magnetometers further highlight that the stable effectiveness of the dynamic eddy-current T-L model hinges on two essential conditions: first, the estimation algorithm must appropriately constrain the coefficient magnitudes; second, the compensation channel should be situated within a cabin environment possessing a sufficiently strong large-scale eddy-current response. Evaluations regarding dual-reference consistency, vector-input sensitivity, and the regularization-parameter sweep confirm that these conclusions remain valid irrespective of the reference definition, the choice of vector magnetometer, or the regularization parameter.

Several limitations of the present study should be acknowledged. First, the improvement is channel-dependent, as a statistically detectable gain is obtained only for the Mag 4 channel under Ridge. Second, the high-dynamic subset contains only five flight lines, so the reported high-dynamic improvement is based on a small sample. Third, the validation is conducted on a single platform from a single public dataset. A dedicated experiment—for example, a scalar magnetometer and a three-axis fluxgate mounted on a controlled test platform, subjected to deliberately designed attitude maneuvers and compared against a ground-based reference magnetometer—would provide more direct evidence of the eddy-current dynamic response, and its implementation is left for future work.

Future work can be effectively directed to take into account operating-condition-dependent effective time constants or extend the eddy-current term to include second- and higher-order dynamic elements to enhance the operating-condition adaptability of the dynamic modification. Moreover, developing an online scheme that adaptively estimates the effective time constant during flight would further improve the practicality of the dynamic modification. Additional directions include validation on other platforms and datasets, as well as a component-level analysis of the vector-magnetometer measurements to provide further insight into the eddy-current response.

## Figures and Tables

**Figure 1:**
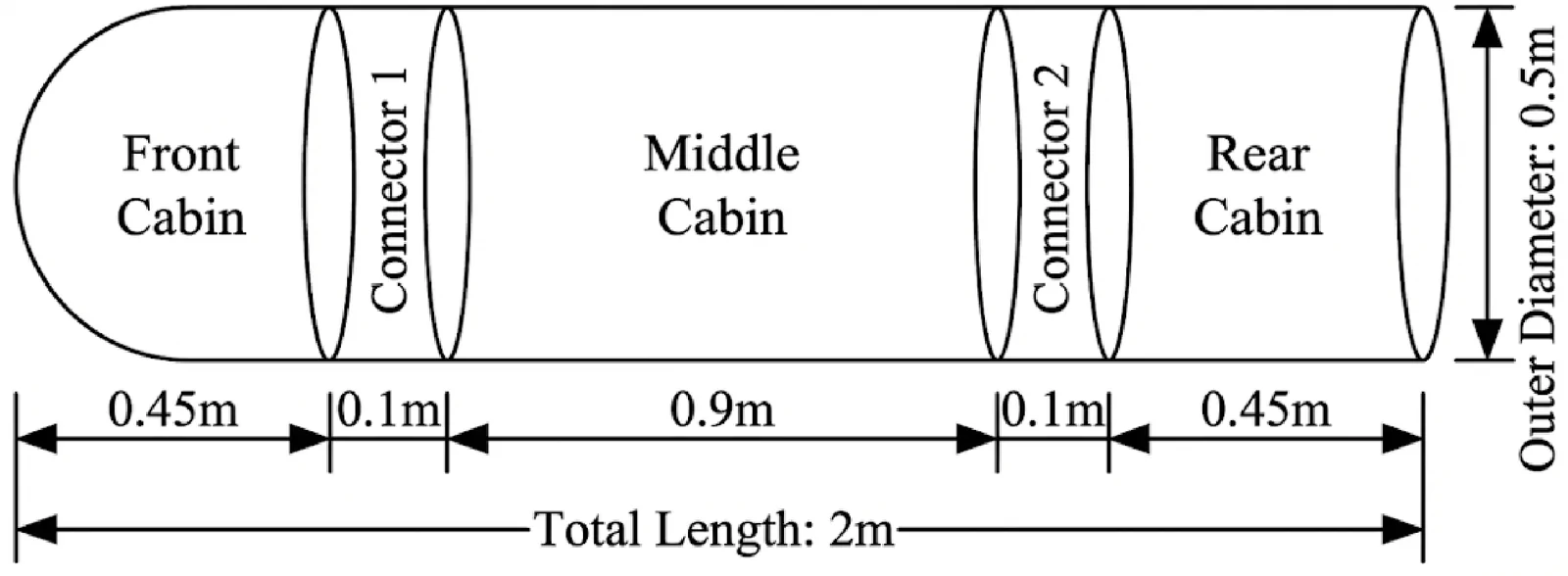
Schematic representation of the simplified model.

**Figure 2:**
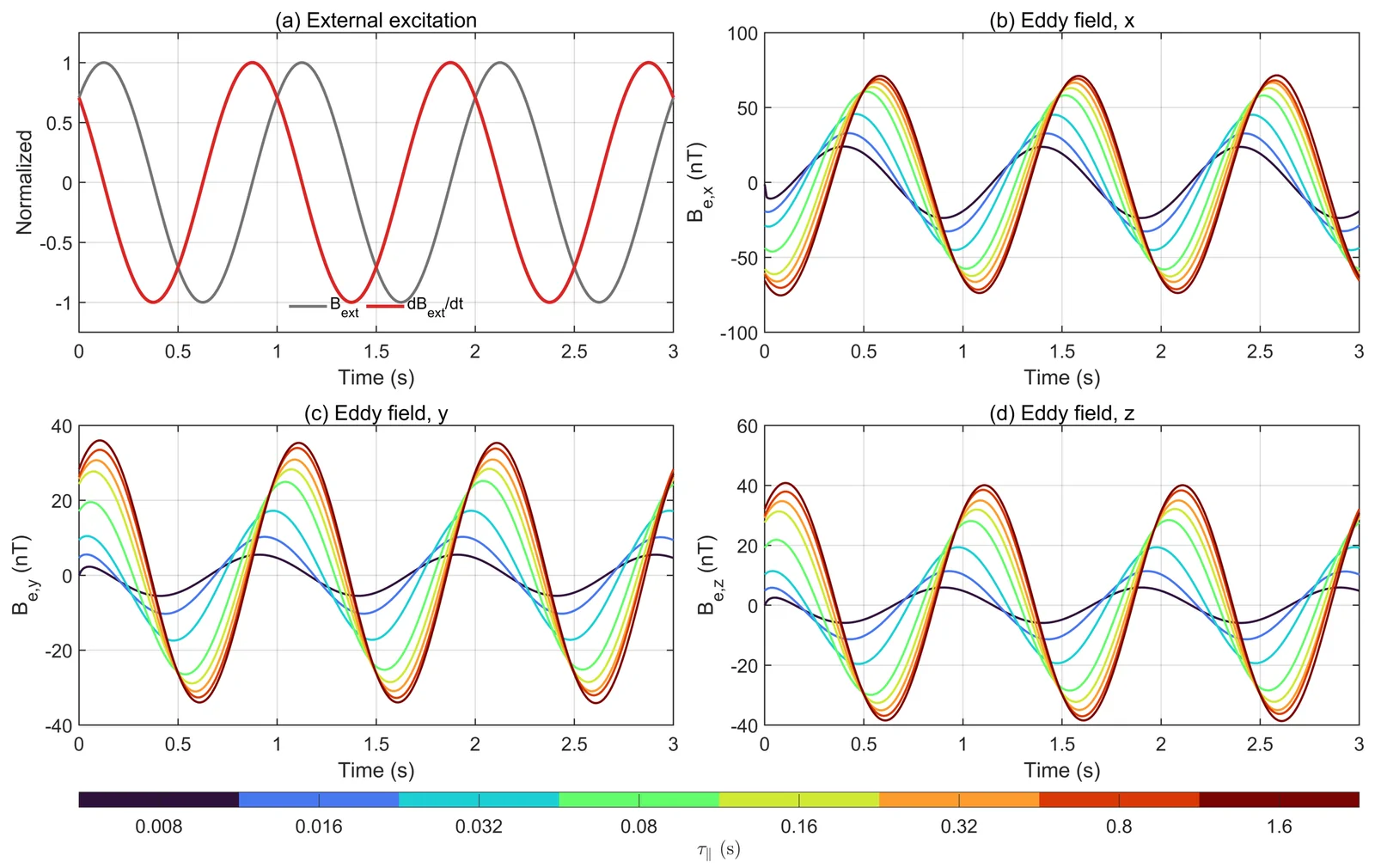
Parametric sweep results for the characteristic time scale.

**Figure 3:**
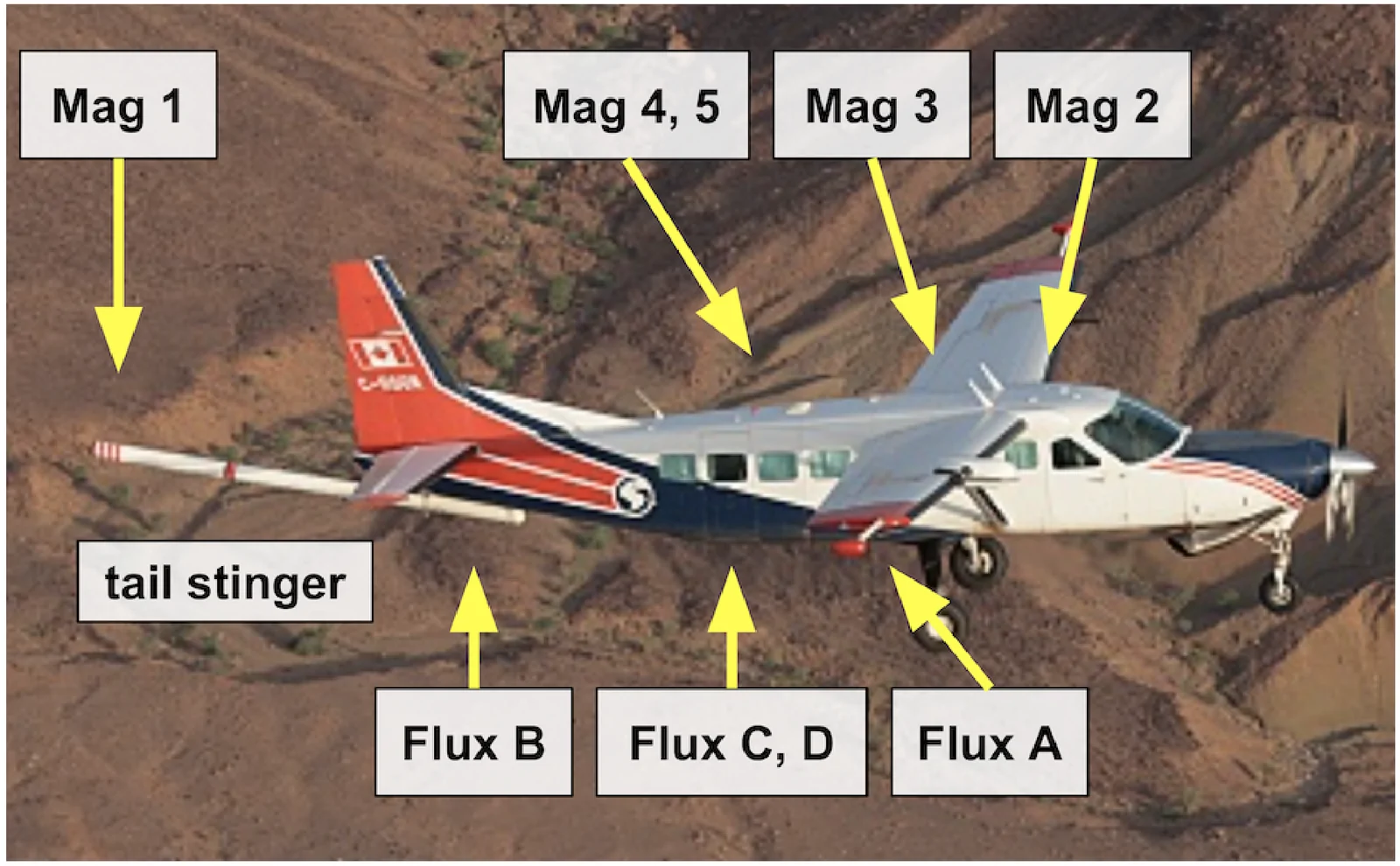
Airborne magnetometer layout, adapted from the public flight dataset [18].

**Figure 4:**
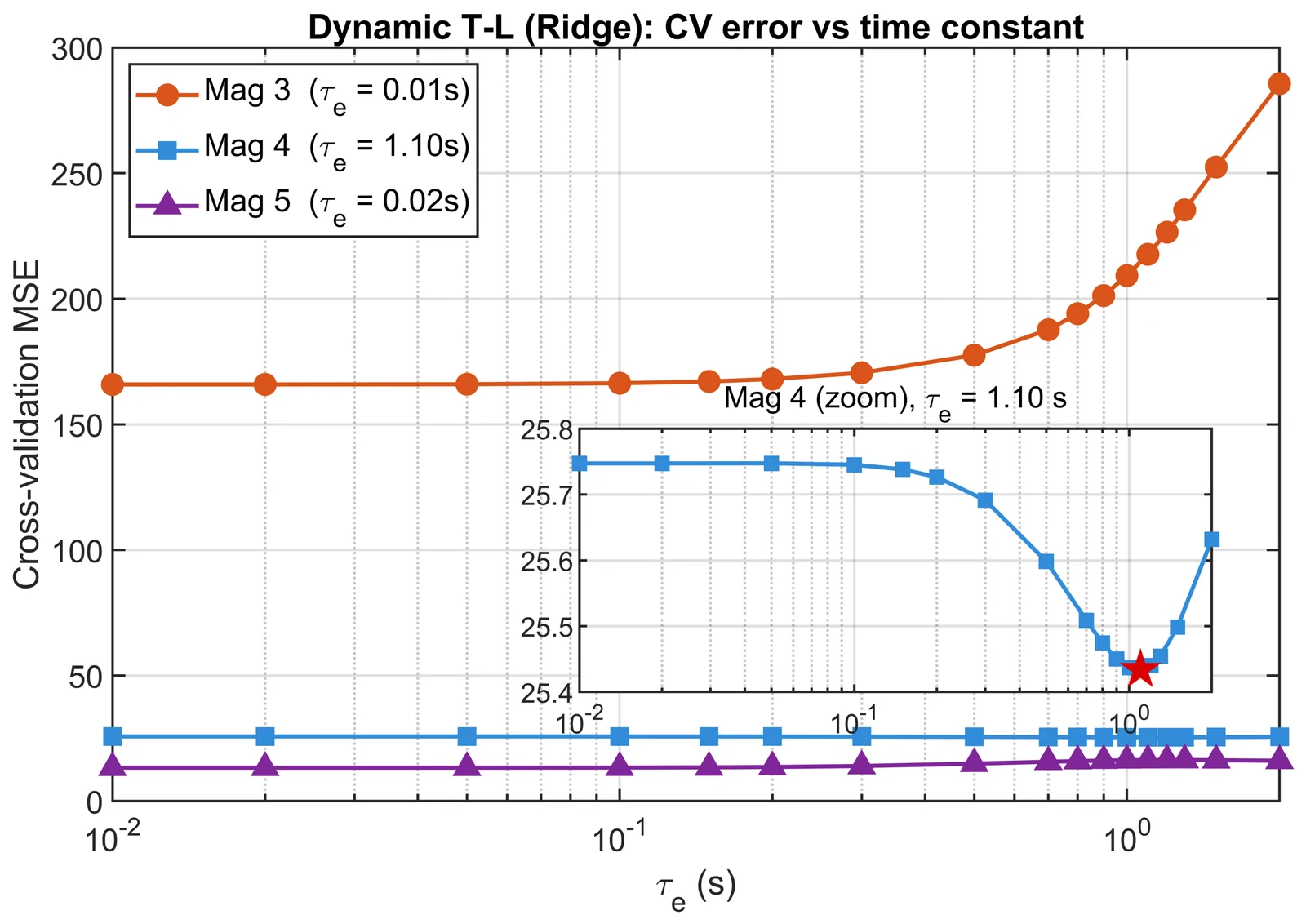
Cross-validation error plots of Mag 3, Mag 4, and Mag 5 based on the Ridge approach. The inset shows an enlarged view of the Mag 4 curve, in which the red star marks the minimum of the cross-validation error and thus the selected effective time constant $\tau_{e}=1.10$ s.

**Figure 5:**
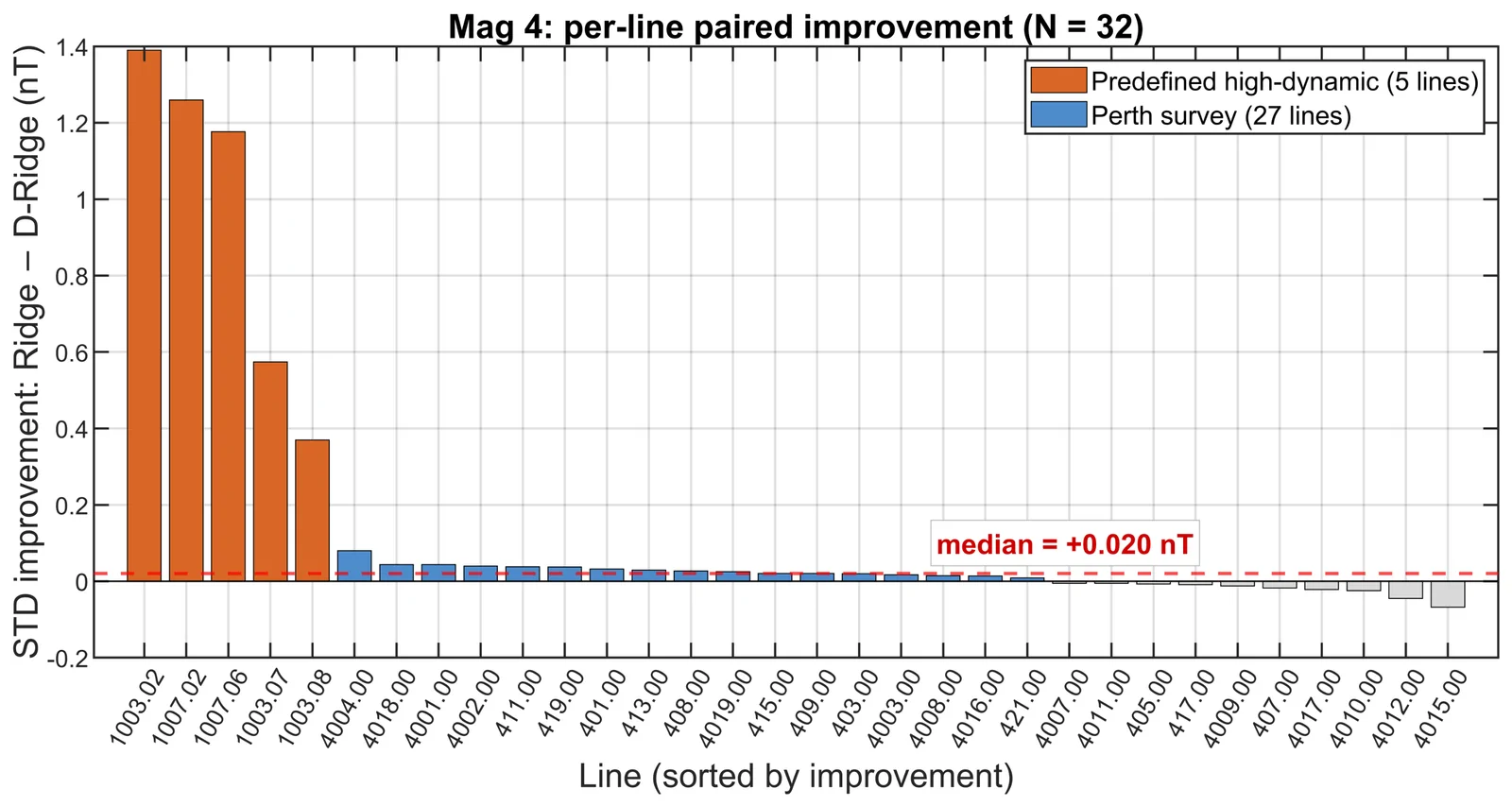
Ranking of Mag 4 paired improvements over the 32 validation flight lines.

**Figure 6:**
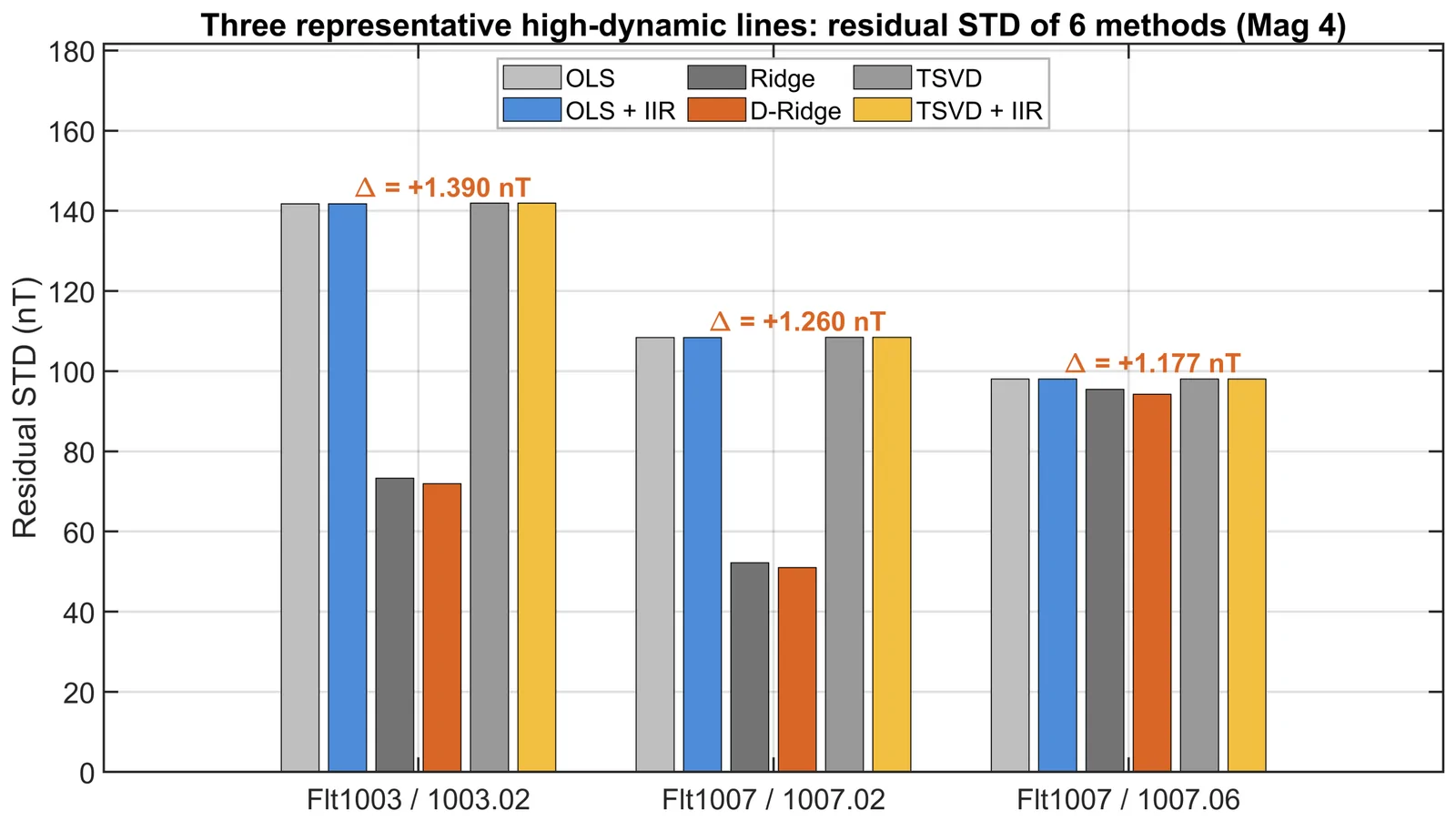
Improvements for three representative high-dynamic flight lines.

**Figure 7:**
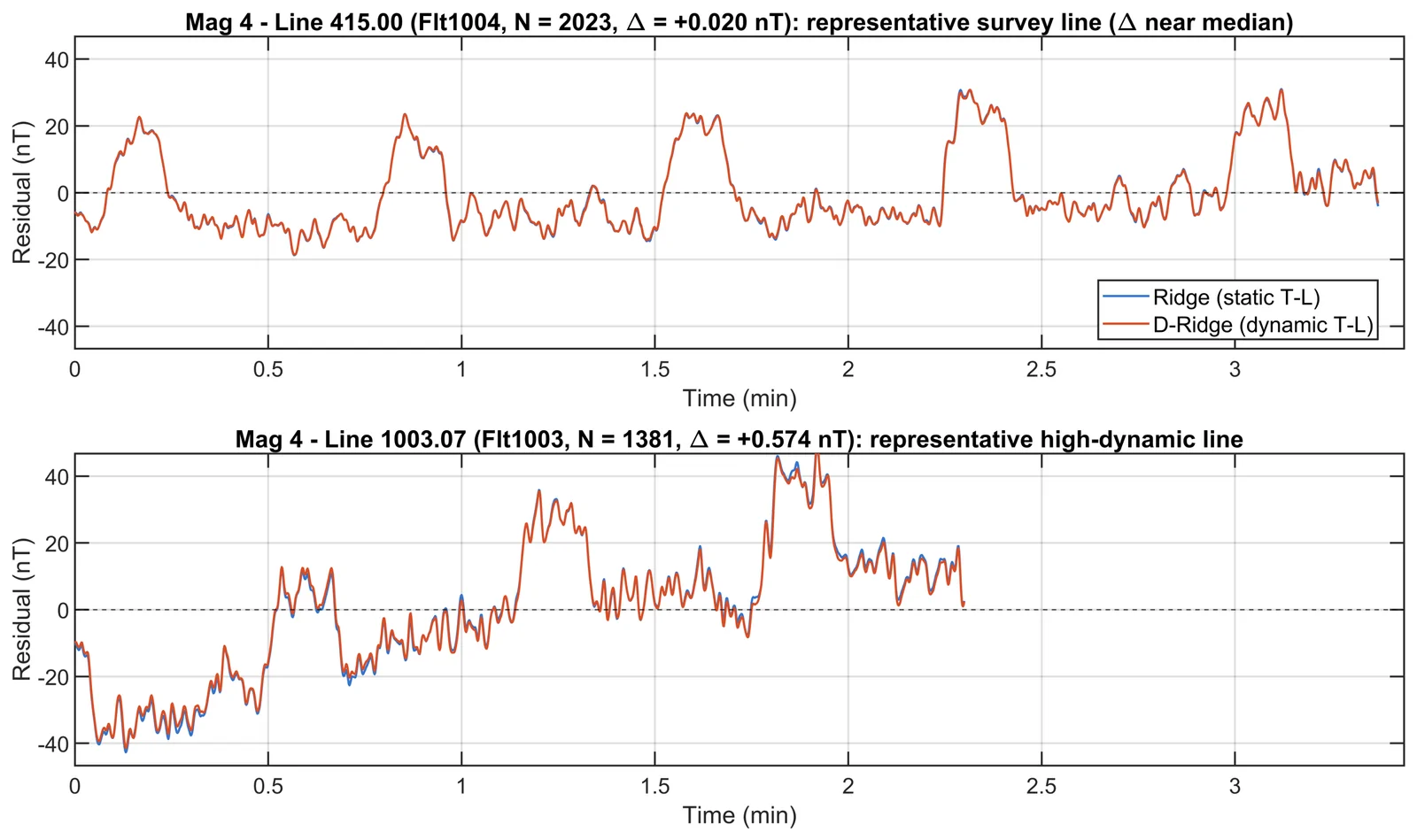
Compensation residuals on representative flight lines: survey line 415.00 in the upper panel and high-dynamic line 1003.07 in the lower panel. The horizontal dashed line in each panel marks the zero-residual reference level.

**Figure 8:**
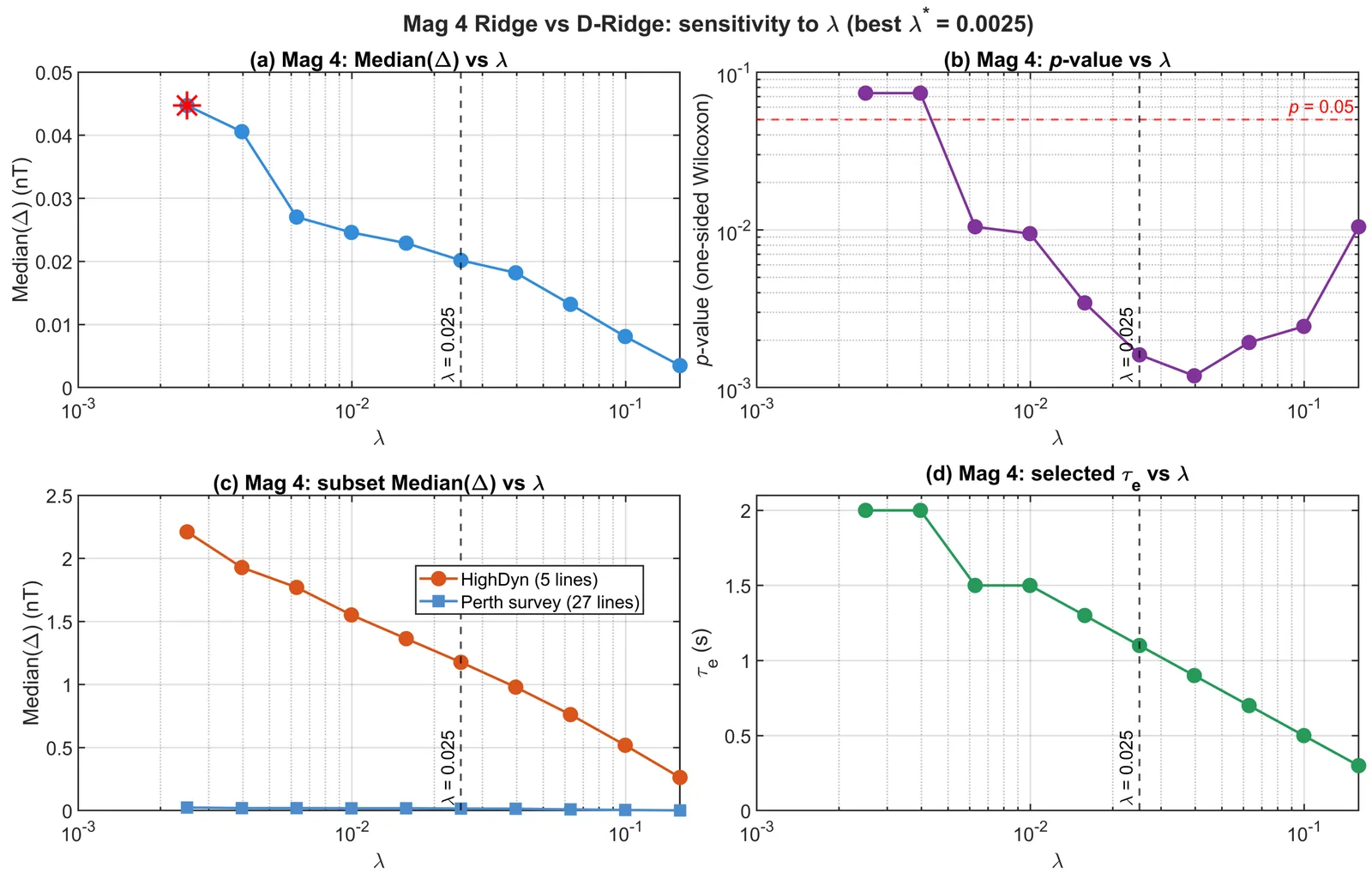
Regularization-parameter sensitivity analysis for the Mag 4 channel: (**a**) median (Δ) vs. λ; (**b**) *p*-value vs. λ; (**c**) subset median (Δ) vs. λ; and (**d**) selected $\tau_{e}$ vs. λ. In panel (**a**), the red asterisk marks the λ value that gives the largest median improvement within the tested grid. In each panel, the black dashed line marks λ=0.025, the value adopted in the main experiment.

**Table 1:** Experimental configuration.

Item	Setting
Data source	DAF-MIT AI Accelerator 2020 SGL public flight dataset; six flight files sampled at 10 Hz
Platform	Cessna Grand Caravan; survey campaign in summer 2020 near Ottawa, Canada
Scalar magnetometers	Five optically pumped, split-beam cesium-vapor sensors (Mag 1–Mag 5); sensitivity 0.005 nT, sensor noise <0.02 nT
Vector magnetometers	Four three-axis fluxgate sensors (Flux A–Flux D)
Attitude source	Three-axis gimballed INS of the AIRGrav system
Primary calibration segment	Flt1006 / TL_i = 6 (Shawville calibration), 6333 samples
Validation set	32 independent validation flight lines from Flt1003, Flt1004, Flt1005, and Flt1007
Maps	Eastern_395, Renfrew_395, Renfrew_555
Compensation targets	Mag 3, Mag 4, Mag 5
Vector input	Flux D for the main experiment; Flux B and Flux C for robustness tests
Reference baselines	Primary baseline: compensated Mag 1 measurement, mag_1_c; auxiliary baseline: map-interpolated value + diurnal correction + International Geomagnetic Reference Field (IGRF)
Excluded data	Official hold-out flight lines 320, 322, 4014, 4013, 4006, 4005, and 1007.04, and all calibration segments; Mag 2 and Flux A are not included in the evaluation
Evaluation metric	Per-line paired comparison of residual standard deviations, with subset-based stratified analysis

**Table 2:** Cross-validation selection results for the OLS and TSVD.

Approach	Mag 3	Mag 4	Mag 5
OLS	0.01 s	0.01 s	0.02 s
TSVD	0.01 s	0.01 s	0.01 s

**Table 3:** Paired statistics for the three channels and three algorithms.

Channel	Algorithm	Median (Δ)	Mean (Δ)	95% CI of Median (Δ)	*p*-Value	Pos Rate
Mag 3	OLS	0.000 nT	0.000 nT	[+0.000, +0.000]	<0.001	96.9%
Mag 3	Ridge	0.000 nT	0.000 nT	[+0.000, +0.000]	0.0937	62.5%
Mag 3	TSVD	0.000 nT	0.000 nT	[+0.000, +0.000]	<0.001	96.9%
Mag 4	OLS	0.000 nT	0.000 nT	[+0.000, +0.000]	1.0000	6.3%
Mag 4	Ridge	+0.020 nT	+0.158 nT	[+0.009, +0.035]	0.0016	68.8%
Mag 4	TSVD	0.000 nT	0.000 nT	[+0.000, +0.000]	1.0000	6.3%
Mag 5	OLS	−0.015 nT	−0.015 nT	[−0.019, −0.012]	1.0000	3.1%
Mag 5	Ridge	0.000 nT	0.000 nT	[+0.000, +0.000]	0.9991	34.4%
Mag 5	TSVD	0.000 nT	0.000 nT	[+0.000, +0.000]	0.8459	43.8%

**Table 4:** Subset-based paired comparison statistics for various ranges.

Range	N	Median (Δ)	Mean (Δ)	*p*-Value
High-dynamic subset	5	+1.177 nT	+0.954 nT	0.031
Regular-survey-line subset	27	+0.017 nT	+0.011 nT	0.024
All flight lines	32	+0.020 nT	+0.158 nT	0.002

**Table 5:** Eddy-current excitation strength of the two subsets, computed as the RMS of the band-pass-filtered rate of change of the Flux D vector field.

Subset	N	Median Excitation (nT/s)	Median (Δ)
High-dynamic subset	5	2134	+1.177 nT
Regular-survey-line subset	27	1278	+0.017 nT

**Table 6:** Consistency of paired improvements under the Mag 1 reference and the map-based reference.

Channel	N	Median (Δ) by Mag 1	Median (Δ) by Map	MeanAbsDiff	MaxAbsDiff
Mag 3	32	+0.000 nT	+0.000 nT	0.0000 nT	0.0000 nT
Mag 4	32	+0.020 nT	+0.024 nT	0.0071 nT	0.0625 nT
Mag 5	32	−0.000 nT	−0.000 nT	0.0001 nT	0.0006 nT

**Table 7:** Sensitivity results for the Mag 4 channel under different vector inputs.

Flux	$\tau_{e}$	Median (Δ)	Mean (Δ)	PosRate
Flux B	1.300 s	+0.006 nT	+0.084 nT	65.6%
Flux C	0.800 s	+0.007 nT	+0.180 nT	59.4%
Flux D	1.100 s	+0.020 nT	+0.158 nT	68.8%

The one-sided Wilcoxon signed-rank test *p*-values for Flux B, Flux C, and Flux D are 0.0418, 0.0592, and 0.0016, respectively.

## Data Availability

The flight data that support the findings of this study are openly available as the DAF-MIT AI Accelerator and Sander Geophysics Ltd. (SGL) public flight dataset [18].
